# Curcumin Ameliorates Cardiac Fibrosis by Regulating Macrophage-Fibroblast Crosstalk *via* IL18-P-SMAD2/3 Signaling Pathway Inhibition

**DOI:** 10.3389/fphar.2021.784041

**Published:** 2022-01-18

**Authors:** Jing Zhao, Yongjian Chen, Qiming Chen, Tingting Hong, Zhiwei Zhong, Junhua He, Cheng Ni

**Affiliations:** ^1^ Department of Cardiology of the Second Affiliated Hospital, Zhejiang University School of Medicine, Hangzhou, China; ^2^ Cardiovascular Key Laboratory of Zhejiang Province, Hangzhou, China; ^3^ Clinical Research Center of the Second Affiliated Hospital, Zhejiang University School of Medicine, Hangzhou, China

**Keywords:** myocardial infarction, curcumin, cardiac fibrosis, interleukin-18, transforming growth factor beta receptor-1

## Abstract

**Ethnopharmacological relevance:** Curcumin is a bright yellow chemical produced by plants of the *Curcuma longa* species. Chemically, curcumin is a diarylheptanoid, belonging to the group of curcuminoids. The therapeutic potential of curcumin has been widely investigated, including its utilization in various of cardiovascular diseases. However, its effect in cardiac remodeling post myocardial infarction and underlying mechanism remains to be uncover.

**Aim:** To evaluate the therapeutic effect and underlying mechanism of curcumin on cardiac fibrosis after myocardial infarction *via* macrophage-fibroblast crosstalk.

**Methods:** Male C57BL/6 (C57) mice were subjected to left anterior descending coronary artery ligation to establish myocardial infarction and intragastrically fed vehicle or curcumin (50 mg/kg or 100 mg/kg) for 4 weeks. In parallel, neonatal rat cardiac fibroblasts were isolated and co-cultured with liposaccharide (LPS^−^ or LPS^+^) curcumin-treated macrophages, followed by TGF-β stimulation for 24 h. Cardiac function was determined by 2-dimensional echocardiography, and cardiac fibrosis was measured by picrosirius red staining. Apoptosis of macrophages was investigated by flow cytometry; all pro-fibrotic protein expression (EDA-Fibronectin, Periostin, Vimentin, and α-SMA) as well as TGF-βR1 downstream signaling activation reflected by phosphorylated SMAD2/3 (*p*-SMAD2 and *p*-SMAD3) were demonstrated by western blotting.

**Results:** Curcumin significantly ameliorated the inflammation process subsequent to myocardial infarction, reflected by decreased expression of CD68^+^ and CD3^+^ cells, accompanied by dramatically improved cardiac function compared with the placebo group. In addition, cardiac fibrosis is inhibited by curcumin administration. Interestingly, no significant reduction in fibrotic gene expression was observed when isolated cardiac fibroblasts were directly treated with curcumin *in vitro;* however, pro-fibrotic protein expression was significantly attenuated in CF, which was co-cultured with LPS-stimulated macrophages under curcumin treatment compared with the placebo group. Mechanistically, we discovered that curcumin significantly downregulated pro-inflammatory cytokines in macrophages, which in turn inhibited IL18 expression in co-cultured cardiac fibroblasts using bulk RNA sequencing, and the TGF-β1-*p*-SMAD2/3 signaling network was also discovered as the eventual target downstream of IL18 in curcumin-mediated anti-fibrosis signaling.

**Conclusion:** Curcumin improves cardiac function and reduces cardiac fibrosis after myocardial infarction. This effect is mediated by the inhibition of macrophage-fibroblast crosstalk in the acute phase post-MI and retrained activation of IL18-TGFβ1-*p*-SMAD2/3 signaling in cardiac fibroblasts.

## Introduction

Cardiovascular diseases, especially ischemic heart disease, remain the leading cause of death worldwide. Although early reperfusion therapy for acute myocardial infarction (MI) can effectively salvage ischemic myocardium, a considerable portion of cardiomyocytes may still experience irreversible necrosis and loss, followed by ventricular remodeling and cardiac insufficiency, which compromises the long-term survival of patients with MI (Tallquist and Molkentin, 2017). Hence, it is crucial to develop effective therapies for adverse cardiac fibrosis subsequent to MI.

**GRAPHICAL ABSTRACT:**
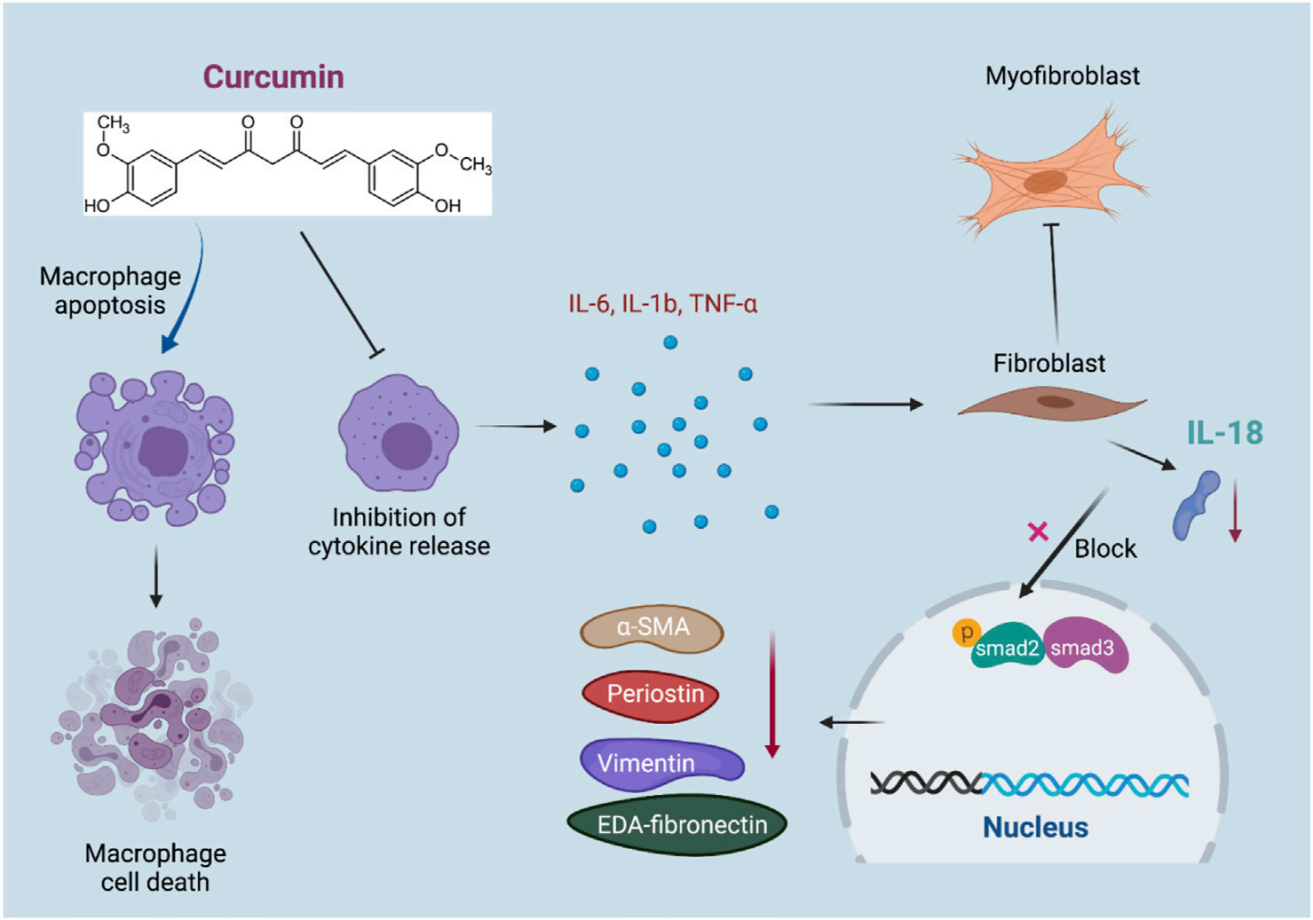


Monocytes and macrophages play a critical role in regulating fibrotic responses in various tissues, including cardiac tissue after ischemic stimulation (Wynn and Vannella, 2016). The normal adult mammalian myocardium contains a relatively small population of resident macrophages (Epelman et al., 2014)^,^ (Heidt et al., 2014) which have been suggested to play a role in cardiac homeostasis by facilitating atrioventricular conduction (Hulsmans et al., 2017). Following injury, resident cardiac macrophages derived from embryonic yolk sac cells are replaced by an abundant population of monocyte-derived macrophages (Epelman et al., 2014), recruited through the activation of chemokine-dependent pathways (Dewald et al., 2003). Macrophages in injured hearts are highly heterogeneous and exhibit functional and phenotypic versatility that enables them to participate in a wide range of processes, including inflammation regulation, fibrosis, matrix remodeling, angiogenesis, and regeneration (Honold and Nahrendorf, 2018). Thus, subsets of activated macrophages may regulate fibrosis by serving as a major source of cytokines and growth factors with fibrogenic properties, secretion of proteases that participate in matrix remodeling, and production of matricellular proteins. Pro-inflammatory cytokines (such as IL-1β, IL-6, and TNF-α) secreted in many cardiac fibrotic conditions may promote a fibrogenic macrophage phenotype by inducing transcription of members of the TGF-β superfamily.

Turmeric is acquired from Curcuma long L, a tuberous herbaceous perennial plant with yellow flowers and wide leaves, which is a member of ginger family and grows in tropical climate (Prasad et al., 2014). Unlike cinnamon, turmeric has not any different kinds. Curcumin is a symmetric molecule consisting of two similar aromatic rings that contain O-methoxy phenolic groups connected by a carbon linker with an α, β-unsaturated β-diketone moiety (Priyadarsini, 2014). Curcumin’s health benefits has been well-established, including anti-tumor, anti-viral, anti-oxidative stress, anti-inflammatory, anti-microbial, hypoglycemic etc. Therapeutically, curcumin exhibits promising potential in preclinical as well as clinical studies and is currently in human trials for a variety of conditions, including metabolic syndrome, nonalcoholic fatty liver disease, atherosclerosis, liver cirrhosis, depression, psoriasis, and Alzheimer’s disease (Kocaadam and Şanlier, 2017). The immunomodulatory functions of curcumin arise due to its interactions with cellular and molecular components during inflammatory reactions. Dietary exposure to 40 mg/kg curcumin for 5 weeks showed enhanced IgG levels in rats, suggesting an improvement in immune function after curcumin intervention (South et al., 1997). Curcumin has also been shown to regulate macrophage polarization by increasing the M2 phenotype marker CD163 together with the anti-inflammatory cytokine IL-10 and decreasing the M1 phenotype marker CD86 along with the pro-inflammatory cytokines TNF-α and IL-6 (Li et al., 2017).

Inflammasomes play an important role in mediating fibrosis in cardiac fibroblasts, which are commonly initiated through NLRP3 activation and response and in turn are amplified by IL-18 secretion (Elliott and Sutterwala, 2015). Due to the increased IL-18 level, the synthesis and secretion of TGF-β1 and cytokines can be promoted through autocrine signaling, leading to further inflammatory activation and phosphorylation of SMAD2/3, which initiates fibrogenesis waterfall reactions.

To summarize, our study aimed to unveil the therapeutic effect of curcumin in alleviating IL-18-*p*-SMAD2/3–induced cardiac fibrosis subsequent to MI, which is mediated by the inhibition of inflammation-induced macrophage-fibroblast crosstalk.

## Materials and Methods

### Chemical Materials

Curcumin (65% purity) and lipopolysaccharide (99% purity) were purchased from Sigma-Aldrich (#C1386 and #L2630, St. Louis, MO, United States), respectively. Recombinant human transforming growth factor-β (TGF-β1, PeproTech, NJ, United States). Raw 264.7 cells were obtained from the American Type Culture Collection (ATCC) (China). Neonatal rat cardiac fibroblasts (NRCFs) were isolated from P0–P3 neonatal rats using the Neonatal Heart Dissociation Kit mouse and rat (#130-098-373) (Miltenyi, United States) in accordance with the manufacturer’s protocols. Dulbecco’s modified Eagle’s medium (DMEM) (#8121348) was obtained from Gibco (Thermo Fisher Scientific, Inc.) Fetal bovine serum (FBS) was obtained from BI (#2053264) (Biological Industries, Inc.), phosphate buffered saline (PBS) (#2104140103), Trypsin (#BC-CE-005-100 ml) and Penicillin/Streptomycin (#BC-CE-007-100 ml) were obtained from (Nanjing BioChannel Biotechnology Co., Ltd, China) and *In Situ* Cell Death Detection Kit, Fluorescein was obtained from Roche Diagnostics (Roche Applied Science, Indianapolis, IN, United States). Annexin V-FITC Apoptosis Detection Kit (#CA1020) was supplied by Beijing Solarbio (Beijing, China). Antibodies against Cleaved-caspase 3 (#9661), *p*-SMAD2 (#3108), and SMAD2/3 (#8685) were purchased from Cell Signaling Technology, Inc. EDA-Fibronectin (#6328), Periostin (#14041), Vimentin (#92547), α-SMA (#5694), *p*-SMAD3 (#52903), CD68 (#125212), CD3 (#16669) and Troponin I (#47003)were purchased from Abcam, Inc. ECL western blot detection kits (#FD8020)**.** were purchased from FD bio Science Biological Technology Co., Ltd (China). A cell counting kit-8 (CCK-8) dye was purchased from Biosharp, Inc. Isoflurane was obtained from RWD Life Science Co., Ltd. Picro Sirius Red (#BP-DL030) was obtained from Sbjbio (Nanjing SenBeiJia Biological Technology Co., Ltd.). ELISA kit was used to quantify IL-18 (#ml002816) (Shanghai Enzyme-linked Biotechnology Co., Ltd.).

### Animals and Experimental Design

Forty male C57/BL/6J mice weighing 20-25 g were purchased from the Zhejiang Experimental Animal Center (Hangzhou, China). All experiments were approved by the Ethical Committee of Zhejiang University and all surgical procedures were performed by experienced technician sin a blinded manner. Mice were acclimatized to the standard conditions with 12 h lighting cycle, 25 ± 2°C temperature, free access to water and standard chow for 1 week. Then, they were sorted into four groups of 10 mice per group. Group 1 (sham) received dimethylsulfoxide (DMSO-saline) for 28 days intragastrically (i.g.) as a vehicle for curcumin. Group 2 (MI + DMOS) was treated with MI.Group 3 was treated with curcumin 50 mg/kg/day i.g. for 28 days after MI. Group 4 was treated with curcumin 100 mg/kg/day i.g. for 28 days after MI. The appropriate dose (50 mg/kg and 100 mg/kg) was selected (Wang et al., 2019; He et al., 2020). MI was induced by ligation of the left anterior descending coronary artery. The MI model was established as described previously (Hu et al., 2008).

### Echocardiograpic Studies

During 4 weeks treatment, mice was anaesthetized (1.5–2% isoflurane mixed with 98% air condition) and transthoracic echocardiography was performed with a 40 MHz transducer (Vevo 2100 Imaging System, VisualSonics, FUJIFILM, Canada). Two-dimensional B-mode and M-mode measurements in the long-axis view level include left ventricular end-diastolic dimension (LVID,d), left ventricular end-systolic dimension (LVID,s), interventricular septal wall thickness in diastole (IVS,d) and in systole (IVS,s), and left ventricular posterior wall thickness in diastole (LVPW,d) and in systole (LVPW,s). Left ventricular ejection fraction and fractional shortening were automatically calculated by the echocardiographic system (Xiao et al., 2018).

### Immunohistochemical Staining

Mouse hearts 7 days post-MI were dehydrated in 30% sucrose solution, embedded in Tissue-Tek OCT compound, snap-frozen in dry ice, and then cut into 7 μm sections. The sections were then stained with a *In Situ* Cell Death Detection Kit (Roche Applied Science, CH), CD3, CD68, Troponin I (Abcam, United Kingdom), and DAPI (Vector Laboratories, Burlingame, CA, United States).

Mouse hearts 28 days post-MI were fixed in 10% formalin-PBS, then paraffin embedded, and cut into 3 μm sections. After deparaffinization, rehydration and tissue antigen recovery, the sections were stained with Periostin (R&D, United States), Troponin I (Abcam, United Kingdom), and DAPI (Vector Laboratories, Burlingame, CA, United States).

### Picro Sirius Red Staining

After deparaffinization, sections were stained with Sirius Red. Tissue damage was scored by calculating the scar circumference, including both internal and external scar diameters. The sirius red–stained sections were scanned with a microscope digital camera (Olympus Instrument, United States), and Biotechnologies, China). The percentage of fibrotic area was calculated as the mean value of the endocardial and epicardial length of the whole fibrotic area in proportion to the mean length of the endocardial and epicardial left ventricle using Image using Image Pro Plus software version 6.0.

### Macrophage Culture and Drug Treatment

Mouse macrophage-like Raw 264.7 cells were acquired from the ATCC and cultured in DMEM with 10% FBS and 1% Penicillin/Streptomycin at 37°C with 5% CO_2_. After 2 days, the medium was replaced, and nonadherent cells were discarded.

### Cytotoxicity Test

Raw 264.7 cell viability was examined using the CCK-8 assay (Bio-sharp, China) in accordance with the manufacturer’s protocols. Cells were treated with liposaccharides (LPS 1 μg/ml) or LPS with curcumin at different concentrations (0.1, 1, 10, 20, and 50 μM) and the appropriate dose (10μM, 20 μM) was selected refer the previous studies (Gao et al., 2015; Chun-Bin et al., 2020). Then Cells were seeded in a 96-well plate at a density of 8×10^3^ cells/well. Following treatment, 10 μL of CCK-8 solution was added to each well and incubated for 2 h. Survival Ratio was calculated according to the following equation: cell survival = [(As-Ab)/(Ac-Ab)] ×100%, where As = treated group, Ac = normal group, and Ab = vehicle control group. The absorbance of each well was measured at 450 and 630 nm using a microplate reader (Spark TECAN, Switzerland). All data were calculated from triplicate samples.

### Cell Apoptosis

Raw 264.7 cells were treated with 1 μg/ml LPS or LPS with curcumin (10 and 20 μM). Cell apoptosis was evaluated by flow cytometry with Annexin V-FITC and PI staining (CytoFlex, Beckman Coulter, Germany). The flow cytometry assay was examined in accordance with the manufacturer’s protocols.

### Cell Co-culture Scheme

NRCFs were isolated from P0–P3 neonatal rats using the Neonatal Heart Dissociation Kit mouse and rat (Miltenyi, United States) in accordance with the manufacturer’s protocols. Raw 264.7 cells were first seeded in 0.4 μm Transwell chamber (#3412), after treatment in low-glucose DMEM with serum deprivation overnight, NRCFs were stimulated with 10 ng/ml TGF-β and then co-cultured with 1 μg/ml LPS or 1 μg/ml LPS with 20 μM curcumin, respectively.

### Plasmid Transfection

NRCFs in each group were seeded in a 6-well plate at 2 × 10^5^ cells per well and cultured in FBS free medium overnight. NRCFs were transfected for 48 h with 2 μg of the IL18 mimic (IL-18 rat, Shanghai GenePharma Co., Ltd, China) to overexpress IL18 using X-tremeGENE HP DNA Transfection Reagent according to the manufacturer’s instructions. Real-time fluorescent quantitative polymerase chain reaction (RT-qPCR) and enzyme-linked immunosorbent assay (ELISA) were used to quantify IL-18 transfection efficiency. Conditioned medium was collected from transfected cell medium and analyzed according to the manufacturer’s instructions.

### Extraction and Quantitative RT-PCR

Total RNA was isolated from three replicates per group after treatment using the TRIzol method, 2 μg of total RNA was reverse-transcribed to cDNA using Evo M-MLV RT Premix for qPCR method according to the manufacturer’s instructions (AG). And resultant cDNA samples were subjected to qPCR on LightCycler^®^ 480 PCR System (Roche) using SYBR^®^ Green Premix Pro Taq HS qPCR Kit (AG). The upstream and downstream primers of IL6, IL-1β, and TNF-α were designed and synthesized by Tsingke Biotechnology Co., Ltd. The primer sequences used were as follows:

Mouse-IL-6, forward (5′-GAG​GAT​ACC​ACT​CCC​AAC​AGA​CC-3′) and reverse (5′-AAG​TGC​ATC​ATC​GTT​GTT​CAT​ACA-3′); mouse-IL-1β, forward (5′-CCA​GCT​TCA​AAT​CTC​ACA​GCA​G-3′) and reverse (5′-CTT​TGG​GTA​TTG​CTT​GGG​ATC-3′); mouse-TNFα, forward (5′-CGG​AGT​CCG​GGC​AGG​T-3′) and reverse (5′- GCT​GGG​TAG​AGA​ATG​GAT​GAA​CA-3′); mouse-GAPDH, forward (5′-TGG​CCT​TCC​GTG​TTC​CTA​C-3′) and reverse (5′- GAG​TTG​CTG​TTG​AAG​TCG​CA-3′); rat-IL-18, forward (5′-TCA​GAC​CAC​TTT​GGC​AGA​CT-3′) and reverse (5′- GAT​TCG​TTG​GCT​GTT​CGG​TC-3′) rat-GAPDH, forward (5′-AAA​GGG​TCA​TCA​CCC​GCC-3′) and reverse (5′- AGT​GAT​GGC​ATG​GAC​TGT​GG-3′).

### Western Blot Analysis

The cells seeded in the 6-well plates were collected, lysed in 2.5×sodium dodecyl sulfate (SDS) gel loading buffer (30 mM Tris-HCl, pH 6.8, 1% SDS, 0.05% bromphenol blue, 12.5% glycerol, and 2.5% mercaptoethanol) and boiled for 30 min; then, 20 μL of the protein was loaded onto 8% concentrated gel for protein electrophoresis separation. After that, the protein was transferred to the PVDF membrane (Millipore, Boston, MA), which was then blocked using 5% skim milk powder for 1 h at room temperature. Then, the membranes were incubated with primary antibodies specific for Cleaved-caspase 3, *p*-SMAD2, SAMD2/3(Cell Signaling Technology), EDA-Fibronectin, Periostin, Vimentin, α-SMA, *p*-Smad3 (Abcam), gapdh (#KC-5G5), and β-actin (#KC-5A08) (KangChen, Shanghai) at 4°C overnight, washed with PBST, and further incubated with a secondary antibody for 1 h. Finally, the immunoreactive protein was detected by a chemiluminescence assay (AI680RGB, GE HealthCare, United States) using the FDbio-Dura ECL kit (FDbio Science Biological Technology Co., LTD, China).

### Statistical Analysis

All results were expressed as value ±standard error of the mean (SEM). Significant differences between two groups were determined by the Student’s t-test, and One-way ANOVA test was used for multiple comparisons, two-way ANOV test was conducted in multiple group with different time points. *p* < 0.05, *p* < 0.01, was considered statistically significant present *, **, respectively. Statistical calculations were carried out using GraphPad Prism 9.0. The sample size was ≥5 in each group for *in vivo* animal studies, and ≥3 in each group for *in vitro* studies.

## Results

### Curcumin Resists Inflammation Response Post-MI and Supports Subsequent Cardiac Function

**FIGURE 1:**
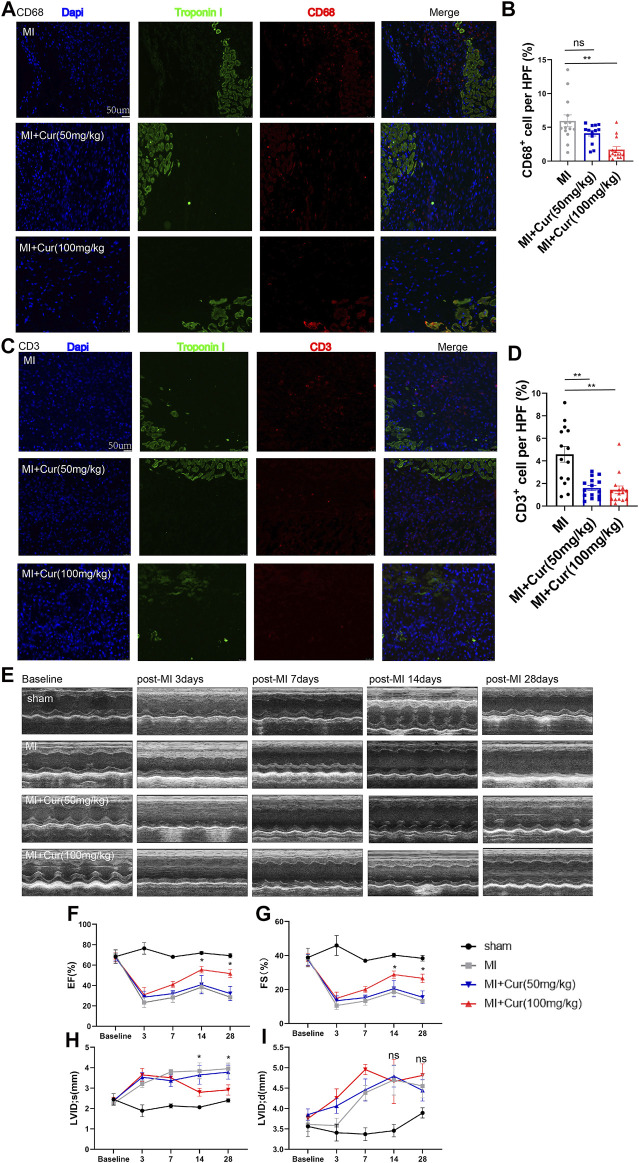
Curcumin administration ameliorates inflammatory response in acute phase post-MI and preserves long-term cardiac function **(A).** Macrophage counts reflected by CD68 staining (red) in peri-infarct heart samples harvested 7 days post-MI from C57 mice in respective group (MI, MI + Cur 50 mg/kg, MI + Cur 100 mg/kg). Troponins were identified by green coloring; statistical data are summarized in **(B).** n = 4-5 in each group **(C).** T cell counts reflected by CD3 staining (red) in peri-infarct heart samples harvested 7 days post-MI from C57 mice (MI, MI + Cur 50 mg/kg, MI + Cur 100 mg/kg). Troponins were identified by green coloring; statistical data are summarized in **(D).** n = 4-5 in each group **(E).** M-mode representative graphs from echocardiography conducted on MI, MI + Cur 50 mg/kg, MI + Cur 100 mg/kg, respectively. Echocardiography were conducted at baseline and 3, 7, 14, and 28 days post-MI. Cardiac function reflected by left ventricle ejection fraction (LVEF%) and left ventricle fraction shortening (LVFS%) in each group (MI, MI + Cur 50 mg/kg, MI + Cur 100 mg/kg) are plotted in **(F)** and **(G)**. n = 5 in each group **(H-I).** Left ventricular internal dimension in systolic phase (LVID s) and Left ventricular internal dimension in diastolic phase (LVID d) in each group at different timepoint were also plotted in **(H)** and **(I)**. MI: myocardial infarction. Results are mean with SEM. NS = no significance between groups, *p < 0.05, **p < 0.01.

To test the effect of curcumin on MI, we performed MI surgery by permanent ligation of the left anterior descending artery followed by intragastric administration of curcumin for 7 days or 28 days. In parallel, a placebo post-MI group was also established. At 7 days post-MI surgery, we discovered significantly inhibited inflammation activation reflected by reduced CD68^+^ and CD3^+^ cells detected in the peri-infarcted area in the curcumin group compared with the placebo group, while no difference in CD68^+^ and CD3^+^ cell counts was observed between the high-dose curcumin group (100 mg/kg) and the low-dose curcumin group (50 mg/kg) (Figures 1A–D)**.** In addition, cardiac function in the MI + curcumin group exhibited significant improvement compared with the placebo group 1 month after MI, with no obvious change observed 7 days post-MI between the two groups, as demonstrated by ejection fraction (EF%) and fraction shortening (FS%) (Figures 1E–G)**,** in addition, the chamber of left ventricle in systolic phase was dramatically decreased in MI + curcumin group compared with MI group (LVID s), while no significant change were detected between groups in diastolic phase (LVID d) (Figures 1H,I). To summarize, the administration of curcumin after MI significantly ameliorated inflammation in the acute phase; however, curcumin exerted a protective effect by preserving long-term cardiac function only after MI, which suggested that the reduced inflammation activation might be related to adverse cardiac remodeling mediated by curcumin intake.

### Curcumin Ameliorates Cardiac Fibrosis and Reverses Adverse Remodeling Post-MI

**FIGURE 2:**
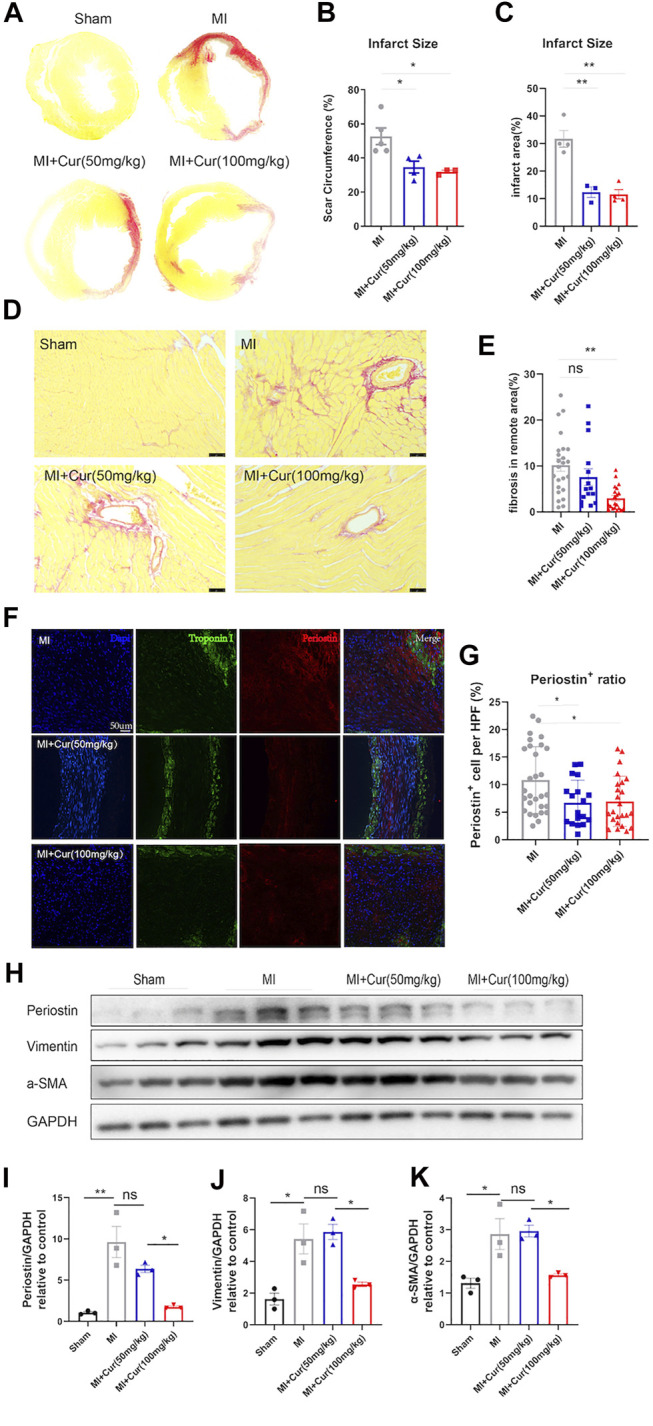
Curcumin reduces scar formation and ameliorates adverse cardiac remodeling in post-MI rats in long-term prospects **(A-B)**. Representative images of sirius red staining of whole heart cross section in rat heart from all groups, as indicated **(A) (B)** Statistical analysis of scar circumference in each group (means with SEM). n = 3–5 per group **(C)** Statistical analysis of infarct area in each group (means with SEM). n = 3–5 per group **(D-E).** Sirius red staining of non-infarcted area in all groups (Sham, MI, MI + Cur 50 mg/kg, MI + Cur 100 mg/kg), scale bar = 100 μm n = 5 per each group **(D)**. Collagen summary and statistics are plotted in **(E) (F-G).** Collagen deposition and content within MI segments measured by Periostin^+^ cells within MI segment; Periostin (red) indicates myofibroblasts that secrete collagen, and Troponin I was stained green. Representative images are displayed in **(F)**, summary data are plotted in **(G)**, n = 4–5 in each group **(H-K)**. Western blot analysis of pro-fibrotic protein expression (Periostin, Vimentin, α-SMA) in each group (Sham, MI, MI + Cur 50 mg/kg, MI + Cur 100 mg/kg), bands are shown in **(H)**, statistics of each protein of Periostin are plotted in **(I)**, Vimentin in **(J)**, α-SMA in **(K)**. Results are mean with SEM, NS = no significance between groups, *p < 0.05, **p < 0.01, n.

As previously indicated, curcumin delivery significantly improved long-term cardiac function after MI in C57 mice. To determine what yields this beneficial effect, we conducted TUNEL and picro sirius red staining and, surprisingly, discovered no significant reduction in anti-apoptotic effect on cardiomyocyte within border area in the curcumin-treated group compared with the placebo group 7 days after MI (Supplementary Figure S1A,B). However, we observed dramatically reduced scar formation reflected by picro sirius red staining in both scar circumference and infarct size dimension in curcumin group compared with the placebo group **(** Figures 2A–C **)**. More importantly, we discovered significant ameliorated fibrosis within non-infarcted area in MI + curcumin group compared with MI group using the dosage of 100 mg/kg (Figures 2D,E), indicating the robust anti-fibrotic role of curcumin post MI. In addition, we conducted immunostaining on MI segments using periostin to verify cardiac myofibroblast enrichment and collagen deposition, revealing significantly decreased periostin^+^ cell expression within MI segment in Curcumin group (Figures 2F,G), western blot results also confirmed the therapeutic utilization of Curcumin in inhibiting pro-fibrotic protein expression as displayed by ameliorated Periostin, Vimentin, and α-SMA expression (Figure 2H–K). The results presented above indicate that the use of curcumin following MI significantly improved cardiac function by inhibiting excessive collagen deposition and scar formation.

### Curcumin Promotes Macrophage Apoptosis Under LPS Stimulation *in vitro* Accompanied by Inhibited Pro-inflammatory Cytokine Secretion

**FIGURE 3:**
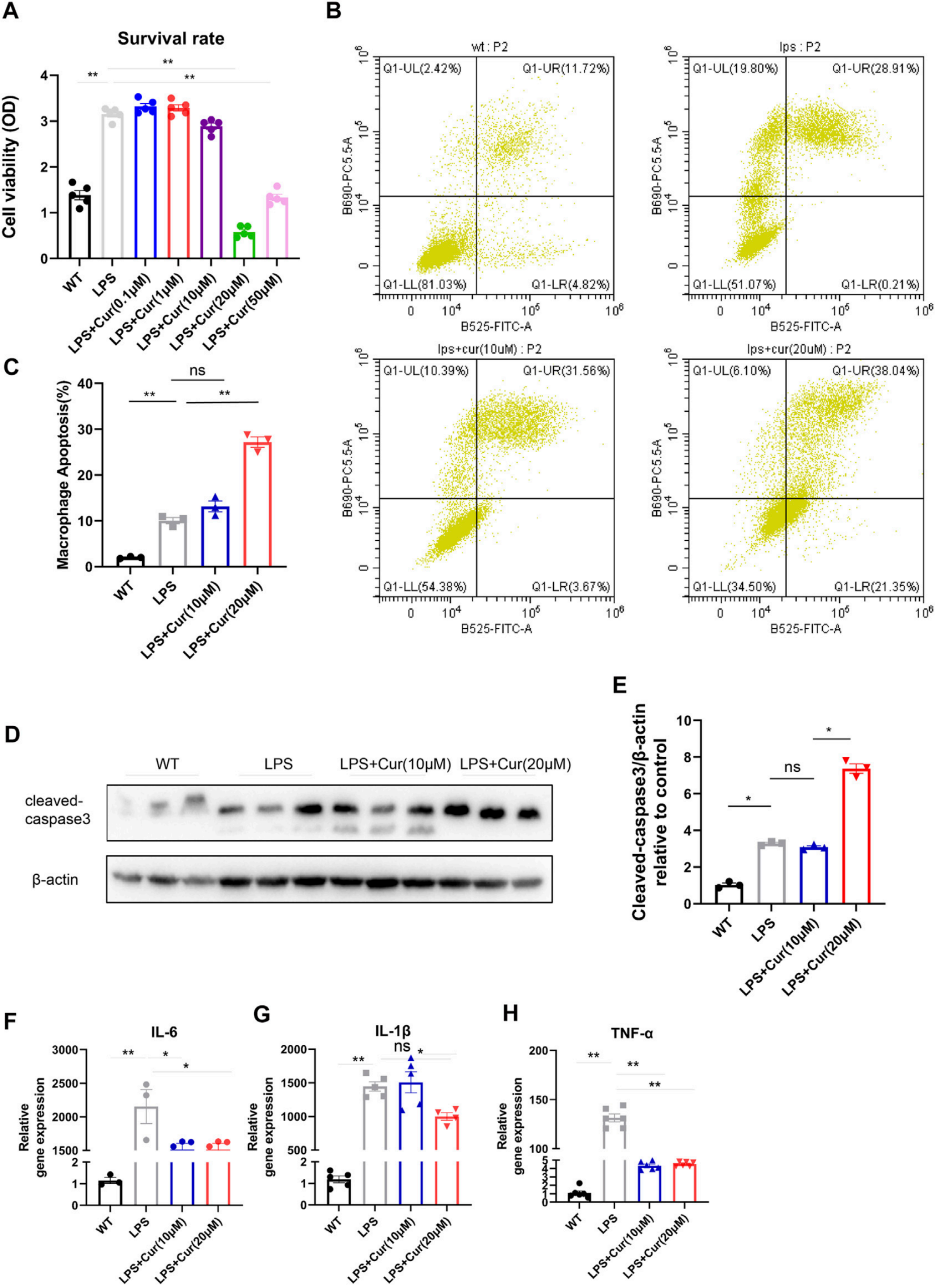
Curcumin promotes macrophage apoptosis *in vitro* and restrain pro-inflammatory cytokine secretio **(A).** Macrophage survival was identified by CCK-8 analysis in respective groups (WT, LPS, LPS + Cur 0.1, 1, 10, 20, and 50 μM) **(B-C)**. Macrophage apoptosis was determined by flow cytometry in respective groups (WT, LPS, LPS + Cur 10 and 20 μM), summary data are displayed in **(C)** **(D**–**E).** Pro-apoptotic protein cleaved-caspase three identification by western blot were conducted in respective groups (WT, LPS, LPS + Cur 10 μM and LPS + Cur 20 μM), original bands are displayed in **(D)** and summary data were illustrated in **(E) (F**–**H).** Pro-inflammatory cytokine detected by qPCR from cell in each group (WT, LPS, LPS + Cur 10 μM and LPS + Cur 20 μM), summary data of IL-6 was plotted in **(F)**, IL-1b in **(G)** and TNF-α in **(H)**. Results are mean with SEM, NS = no significance between groups, *p < 0.05, **p < 0.01.

It is known that LPS significantly activate inflammation and mobilize macrophage proliferation as well as M1 polarization. To further investigate the mechanism of curcumin in anti-inflammatory processes, we performed LPS stimulation on macrophage *in vitro* for 24 h followed by Curcumin administration, CCK8 test revealed dramatic inhibited macrophage proliferation when dosage was 20 uM, indicating Curcumin might exerted anti-proliferation or pro-apoptotic effect in macrophages under LPS stimulation (Figure 3A). Flow cytometry was conducted to determine whether curcumin promoted macrophage apoptosis under LPS stimulation. As shown in Figures 3B,C, curcumin significantly increased macrophage apoptosis under LPS stimulation compared with LPS only also using 20 uM dosage. In addition, the cleaved-caspase three protein level, which acts as a marker of apoptosis, was also significantly up-regulated in the curcumin-treated macrophages with LPS group compared with LPS only reflected by western blot (Figures 3D,E). Furthermore, macrophage activation could induce the secretion of pro-inflammatory cytokines such as IL-6, IL1β, and TNF-α to initiate an immune response to ischemic injury, thus aggravating cardiomyocyte apoptosis; however, curcumin treatment significantly inhibited pro-inflammatory cytokine secretion in macrophages with LPS group compared with LPS only, as reflected by reduced IL-6, IL-1β, and TNF-α secretion especially at 20 uM concentration (Figures 3F–H). These data indicate that the administration of curcumin on macrophages under LPS stimulation exerted both pro-apoptotic effects in macrophages and anti-inflammatory effects by reducing cytokine release from macrophages, which could be greatly beneficial post-MI.

### Curcumin Exerts Anti-Fibrotic Effects via Macrophage-Fibroblast Crosstalk

**FIGURE 4:**
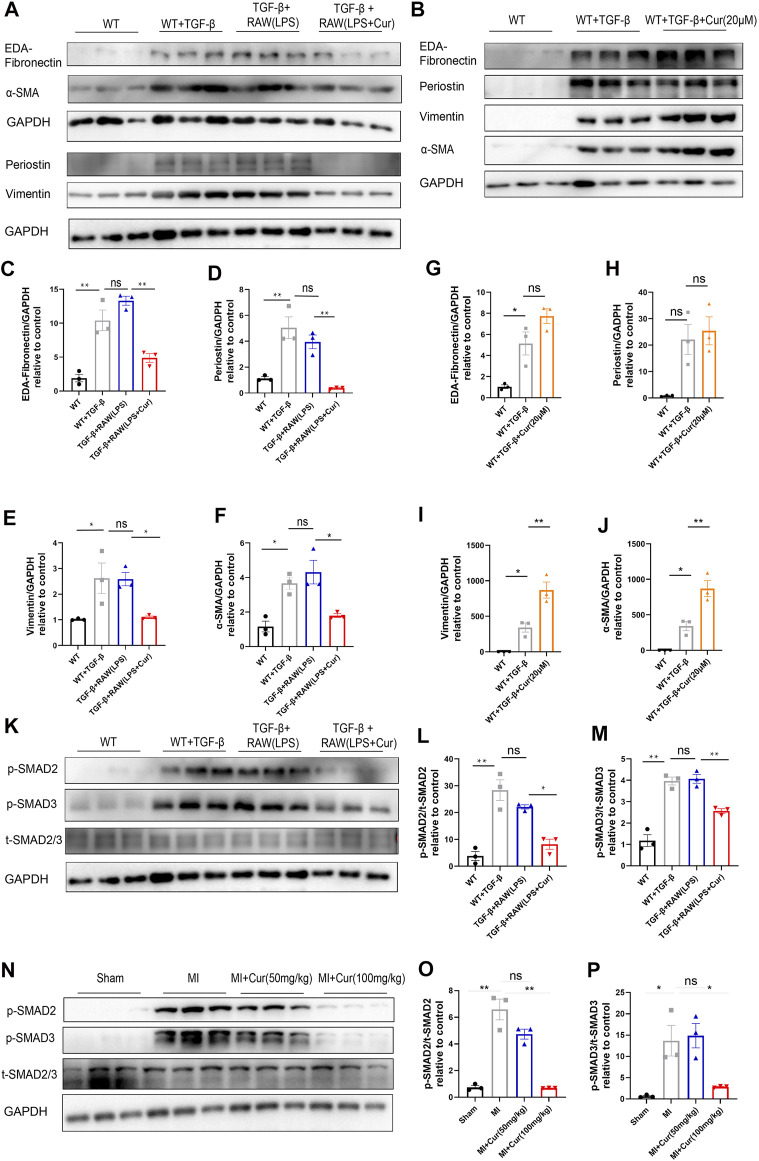
Curcumin inhibits cardiac fibrosis by regulating macrophage-fibroblast crosstalk instead of directly suppressing cardiac fibroblast *trans*-differentiation **(A).** Fibrotic protein expression in different NRCF group (WT, WT + TGF-β, TGF-β+RAW (LPS), TGF-β+RAW (LPS + Cur)) respectively, fibrotic protein is defined as EDA-Fibronectin, periostin, vimentin and α-SMA **(B).** Fibrotic protein expression in different NRCF group (WT, WT + TGF-β, WT + TGF-β+Cur) respectively **(C-F).** Summary data of protein expression are displayed in **(A)**, EDA-fibronectin are plotted in **(C)**, periostin is plotted in **(D)**, vimentin is plotted in **(E)** and α-SMA is plotted in **(F) (G**–**J)**. Summary data of protein expression displayed in **(B)**, EDA-Fibronectin was plotted in **(G),** periostin was plotted in **(H)**, vimentin is plotted in **(I)** and α-SMA in **J) (K**–**M).** TGF-βR1 downs-stream signaling identification by western blot in each group (WT, WT + TGF-β, TGF-β+RAW (LPS), TGF-β+RAW (LPS + Cur)); phosphorylated SMAD2 and SMAD3 were determined as key factors underlying TGF-βR1 signaling to promote fibrosis procedure. Western blot bands are displayed in **(K)**, and summarized data are plotted in **(L-M)** (**N-P)** Phosphorylation level of SMAD2/3 in respective groups (Sham, MI, MI + Cur 50 mg/kg, MI + Cur 100 mg/kg) 1 month after MI were detected by western blotting, bands are shown in **(N)**, and summarized data are plotted in **(O**–**P)**. Results are mean with SEM, NS = no significance between groups, *p < 0.05, **p < 0.01.

As previously mentioned, curcumin has also been shown to regulate macrophage polarization by increasing anti-inflammatory cytokine IL-10 levels and decreasing the M1 phenotype marker CD86 along with the pro-inflammatory cytokines TNF-α and IL-6. To validate whether curcumin exerted an inhibitory effect on cardiac fibrosis by alleviating inflammation-induced fibrosis or by directly suppressing cardiac fibroblast *trans*-differentiation and collagen secretion, we co-cultured macrophages under LPS stimulation with isolated primary neonatal rat cardiac fibroblasts *in vitro* followed by TGF-β stimulation in NRCF for 24 h, Surprisingly, we discovered that curcumin administration in macrophages with LPS stimulation significantly mitigated collagen synthesis from co-cultured NRCF, which was revealed by pro-fibrotic protein expression (α-SMA, Vimentin, Periostin, and EDA-Fibronectin) (Figure 4A,C–F). In contrast, we did not observe a significant change in fibrosis protein expression when curcumin was directly added to NRCF treated with TGF-β for 24 h compared with the TGF-β only group (Figure 4B,G–J). Mechanistically, we discovered decreased phosphorylation of SMAD2/3 in the curcumin-macrophage co-cultured NRCF group compared to NRCF without curcumin treatments, as reflected by the *p*-SMAD2/3 to total SMAD2/3 ratio (Figure 4K–M), importantly, we also discovered down-regulated phosphorylation of SMAD2/3 in hearts from post-MI with curcumin treated group compared with MI group, indicating the consistent role in anti-phosphorylation of curcumin towards SMAD2/3(Figure 4N–P). In summary, the administration of curcumin only inhibited LPS-stimulated macrophage-fibroblast crosstalk induced excessive collagen deposition, this effect is mediated by the inhibition of SMAD2/3 phosphorylation, while in NRCF with TGF-β stimulation, curcumin delivery was unable to reverse established pro-fibrotic protein expression.

### Curcumin Alleviates Cardiac Fibroblast Trans-differentiation by Inhibiting IL-18 Expression Promoted by Macrophage-Fibroblast Crosstalk

**FIGURE 5:**
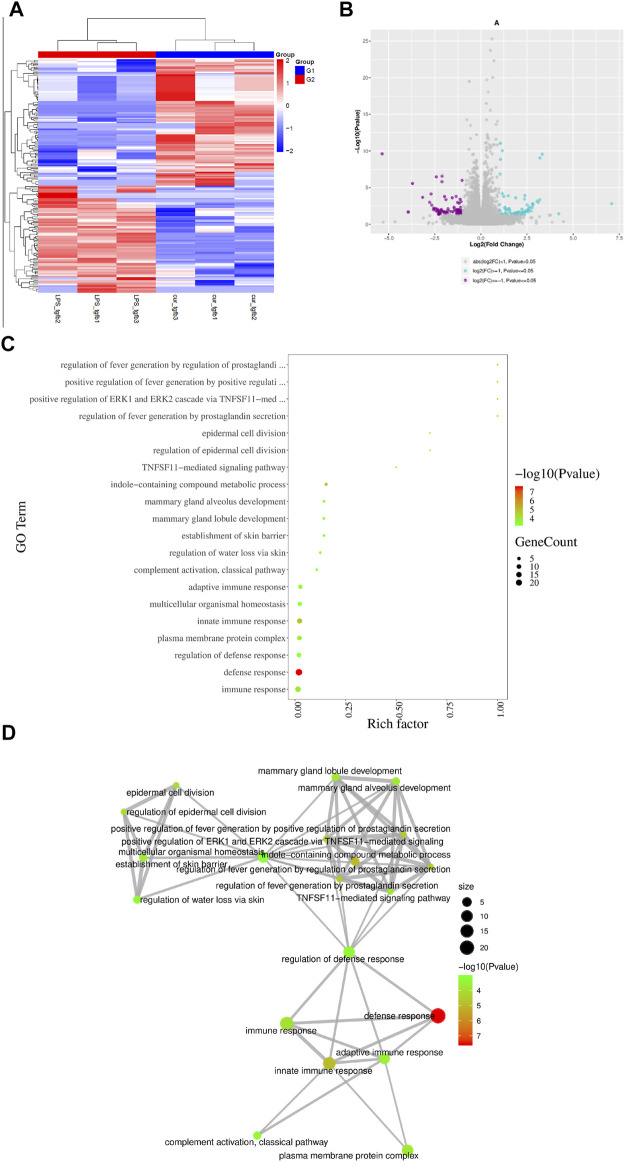
IL-18 plays significant role in curcumin-mediated anti-fibrotic effect screened from mRNA sequencing **(A).** Difference gene heat map between two NRCF group (TGF-β+RAW (LPS), TGF-β+RAW (LPS + Cur)). N = 3 in each group **(B).** Volcano plots indicating up-regulated genes and down-regulated genes between two groups. Purple dots represent down-regulated genes, blue dots represent up-regulated genes, and grey dots represent no significant change. **(C**–**D).** GO signaling pathway enrichment analysis summary from respective groups. Different colors and dot sizes indicate gene number and gene alteration, respectively. Mutual correlation among all signaling enrichment observations are displayed in **(D)**.

To elucidate the detailed mechanism of the identified curcumin-mediated anti-fibrosis effect, we performed bulk mRNA sequencing in NRCF co-cultured with curcumin-treated macrophages and NRCF co-cultured with PBS-treated macrophages; 776 genes were downregulated and 1,467 genes were upregulated in the curcumin group compared with the PBS group (Figures 5A,B). Gene enrichment from GO pathway analysis revealed that both immune and defense response signaling were mostly activated and altered between the two groups and that these two signaling pathways were classified and regulated by defense response regulation, indicating the pivotal utilization of curcumin in alleviating an overreactive immune response (Figures 5C,D), the top 10 up-regulated and down-regulated genes enriched in immune response signaling are listed in Table 1. Among all altered genes enriched in immune response signaling, IL-18 was found to be the top-ranked gene that was significantly down-regulated in the curcumin group, considering the established pro-fibrotic effect mediated by inflammosome-secreted IL-18 and NLRP3 activation. We speculated that the anti-fibrosis effect exerted by curcumin was mediated by mitigating IL-18 expression and secretion in cardiac fibroblasts.

**TABLE 1:** Top 10 up and down-regulated genes enriched in inflammation response pathway. (NRCF, co-cultured with PBS-treated macrophages with TGF-β, administration) vs (NRCF, co-cultured with curcumin-treated macrophages with TGF-β, administration).

Gene ID	Gene symbol	FDR	Fold of change log2 (NRCF vs NRCF + Cur)	Up-down
ENSRNOG00000015615	Tnfrsf11a	0.034	3.197800686	up
ENSRNOG00000009848	IL-18	0.035	2.94645754	up
ENSRNOG00000009471	Epsti1	0.014	2.812906485	up
ENSRNOG00000006314	Zbp1	0.043	2.79906252	up
ENSRNOG00000017606	P2rx1	0.0024	2.720746936	up
ENSRNOG00000018798	Bcan	0.025	2.66189736	up
ENSRNOG00000025603	Adgrf2	0.0017	2.581454723	up
ENSRNOG00000001923	Tprg1	0.043	2.558506757	up
ENSRNOG00000010270	Espn	0.036	2.548806342	up
ENSRNOG00000017602	IL-34	0.025	2.386965316	up
ENSRNOG00000020492	Ubbp4	0.0098	−5.355885393	down
ENSRNOG00000036622	Glyatl1	0.012	−2.777779252	down
ENSRNOG00000026296	Saxo2	0.0086	−2.569574924	down
ENSRNOG00000008807	Rp1	0.021	−2.561298476	down
ENSRNOG00000028404	Ppp1r1b	0.0015	−2.430345144	down
ENSRNOG00000010263	Cldn11	0.036	−2.365653939	down
ENSRNOG00000009269	Cga	0.0142	−2.325447584	down
ENSRNOG00000021039	Fam83e	0.048	−2.320912344	down
ENSRNOG00000011334	Tmem63c	0.039	−2.279315173	down
ENSRNOG00000012906	Bcas1	0.017	−2.254907096	down

### IL-18 Overexpression Neutralizes Anti-Fibrotic Effect in NRCF Co-cultured With Curcumin-Treated Macrophage

**FIGURE 6:**
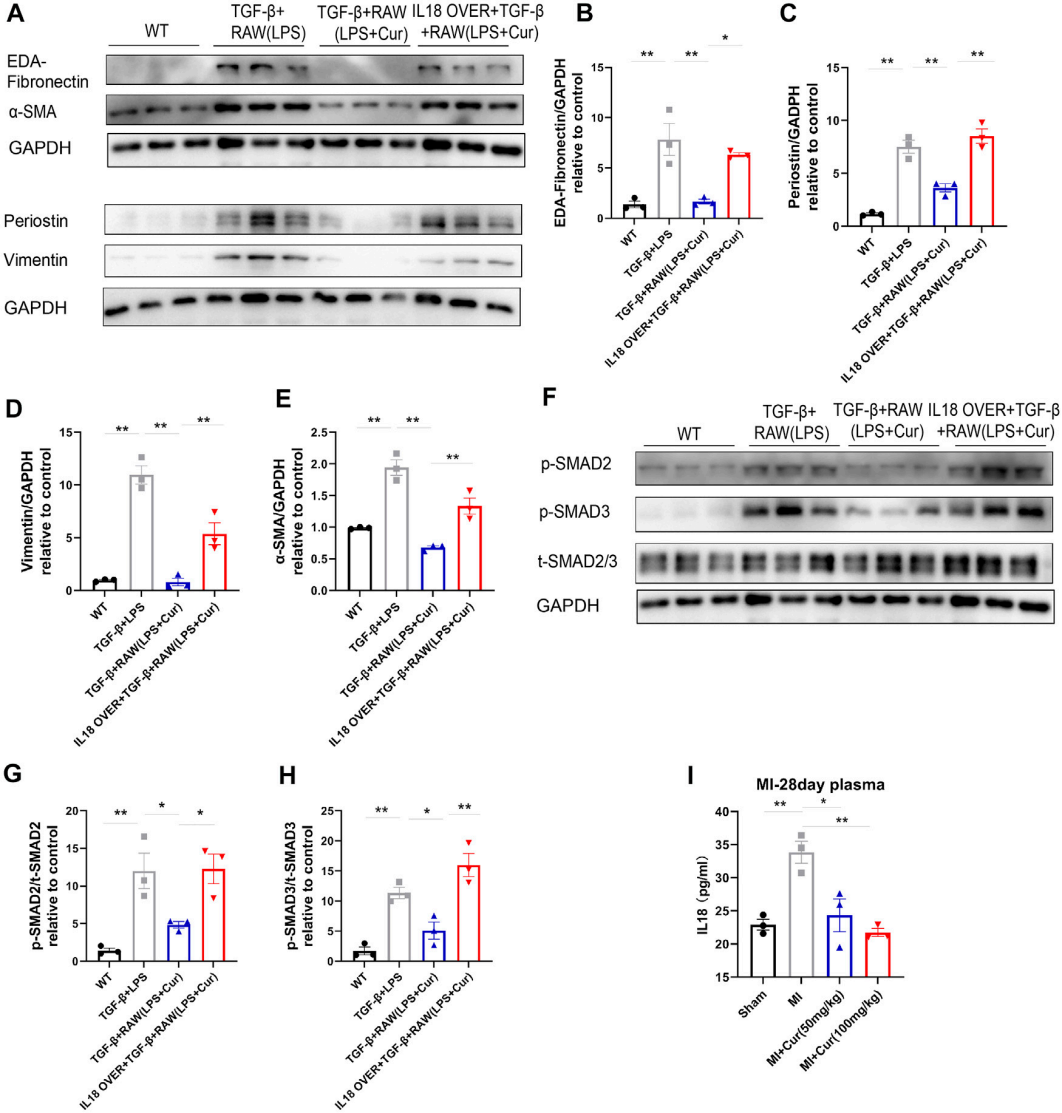
IL-18 compensation represses curcumin-induced inhibition of SMAD2/3 phosphorylation and fibrotic gene expression **(A**–**E).** Fibrotic protein expression in different NRCF groups (WT, TGF-β+RAW (LPS), TGF-β+RAW (LPS + Cur), IL18 over + TGF-β+RAW (LPS + Cur)) respectively, fibrotic proteins are defined as EDA-Fibronectin, Periostin, Vimentin, and α-SMA. Summary data of protein expression are displayed in **(B**–**E)**. EDA-Fibronectin is plotted in **(B),** Periostin is plotted in **(C)**, Vimentin is plotted in **(D)** and α-SMA in **(E)** **(F-H).** Phosphorylation level of SMAD2/3 in different NRCF groups (WT, TGF-β+RAW (LPS), TGF-β+RAW (LPS + Cur), IL18 over + TGF-β+RAW (LPS + Cur)) identified by western blot is illustrated in **(F)**. Summary data are plotted in **(G)** for *p*-SMAD2 and **(H)** for *p*-SMAD3 **(I).** IL-18 content in plasma from different groups (Sham, MI, MI + Cur 50 mg/kg, MI + Cur 100 mg/kg) were identified by ELISA kit, summary data are plotted. Results are mean with SEM, NS = no significance between groups, *p < 0.05, **p < 0.01.

Previously, we identified IL-18 as a central molecule in mediating LPS-treated macrophage-activated myofibroblast *trans*-differentiation on NRCF, while curcumin administration significantly ameliorated this process. Hence, to test whether compensation of IL-18 in NRCF after co-culture reverses the anti-fibrotic effect of curcumin, we constructed an IL-18 overexpression plasmid and transfected it into TGF-β-stimulated NRCF after co-culture with LPS and curcumin double-treated macrophages. We discovered significantly upregulated IL-18 gene expression and considerable transfection efficacy, as demonstrated by RT-PCR and immunofluorescence (Supplementary Figure S2A,B). Furthermore, IL-18 protein expression levels were determined by ELISA in the supernatant collected from NRCF, indicating successful overexpression after plasmid transfection (Supplementary Figure S2C). As expected, IL-18 overexpression in NRCF significantly reversed the curcumin-mediated anti-fibrotic effect, as shown by the expression of fibrotic genes (i.e., EDA-Fibronectin, Periostin, Vimentin, and α-SMA) were detected by western blot analysis (Figure 6A–E) more importantly, SMAD2/3 phosphorylation, which was inhibited by curcumin treatment, rebounded by IL-18 overexpression in NRCF (Figure 6F–H). Furthermore, IL-18 content was also discovered to be significantly down-regulated in plasma from MI + curcumin group compared with MI group using ELISA, which is consistent with the outcome from unbiased transcriptome analysis as well as *in vitro* findings above (Figure 6I). From these findings, we conclude that IL-18-*p*-SMAD2/3 signaling plays a critical role in the curcumin-mediated anti-fibrosis mechanism in TGF-β-stimulated NRCF co-cultured with LPS-treated macrophages.

## Discussion

Cardiac fibrosis, characterized by intersection and collagen deposition within the cardiac interstitium due to net accumulation of extracellular matrix (ECM) proteins, is a common pathophysiological manifestation in most myocardial diseases (Berk et al., 2007)^,^ (Kong et al., 2014). In general, the extent of fibrotic remodeling is closely associated with adverse organ outcomes. Myocardial fibrosis is not necessarily the primary cause of dysfunction. In many circumstances, cardiac fibrosis is the result of a reparative process that is activated in response to cardiomyocyte injury. In humans or other adult mammals, quiescent fibroblasts sustained dynamic balance with cardiomyocytes in healthy status; however, these cells have strong potential in repairing injured myocardium; thus, after pathological conditions, loss of a significant number of cardiomyocytes triggers a reparative program, leading to the formation of fibrous tissue. For example, in acute MI, the sudden death of many cardiomyocytes initiates an intense inflammatory reaction, ultimately leading to the replacement of dead myocardium with a collagen-based scar (Frangogiannis, 2012).

Cardiac fibrosis in response to ischemic injury can be divided into three stages: acute early response, proliferation, and late maturation. Inflammation plays a pivotal role in the acute early response, which initiates subsequent repair and acts as a “sentinel.” Macrophages are activated immune-cells in early phase post infarction that secrete various cytokines and are intimately correlated with the activation of cardiac fibroblast, initiating “macrophage-fibroblast crosstalk.” Pro-inflammatory activation of cardiac fibroblasts is associated with inflammasome induction, which leads to caspase stimulation and IL-1β secretion by macrophages (Kawaguchi et al., 2011)^,^ (Sandanger et al., 2013). In cultured rat heart fibroblasts, higher gelatinase activity was observed in response to stimulation with IL-1β, and tumor necrosis factor α (TNF-α). Interleukin-1β and tumor necrosis factor alpha decreased collagen synthesis and increased matrix metalloproteinase activity in cardiac fibroblasts *in vitro*, and they also differentially regulated the production of tissue inhibitors of metalloproteinases. After ischemia reperfusion–induced heart injury, IL-1 receptor 1-deficient mice showed decreased accumulation of macrophages in the infarcted myocardium and diminished early inflammatory and pro-fibrotic responses (Bujak et al., 2007) suggesting downregulation of cardiac repair in a case of disrupted IL-1-dependent signaling. IL-1β inhibits fibroblast proliferation by inducing cell cycle arrest during the G1/S transition (Palmer et al., 1995). However, extended exposure of cardiac fibroblasts to inflammatory cytokines, such as IL-1β, was observed in the prolonged inflammatory phase post-MI. The long-term action of IL-1 β on cardiac fibroblasts delays or prevents the transition from the pro-inflammatory stage to the proliferative phase of heart repair, which may induce adverse remodeling and heart failure by reducing heart contractility and promoting cardiomyocyte apoptosis (Bujak and Frangogiannis, 2009). In contrast to IL-1β, TNF-α may indirectly induce the profibrotic activity of fibroblasts by increasing the expression of type 1 angiotensin II (AT1) receptors. Indeed, most inflammatory cytokines that cooperate in the pro-inflammatory activation of cardiac fibroblasts can demonstrate effects on fibrotic fibroblast activity. In our study, we revealed that macrophages secreting IL-1β, TNF-α, and IL-6 play a significant role in mediating cardiac fibroblast *trans*-differentiation, which is in accordance with the conclusions derived from the aforementioned previous studies.

Curcumin has roles in various cardiovascular diseases, including ischemic heart, pressure overload heart, and metabolic disorder-related cardiac diseases. It is well-established that curcumin can directly exerts cardio-protective effect by targeting cardiomyocyte through various of signaling pathway, like disrupts the p300/GATA4 complex and represses agonist-and p300-induced hypertrophic responses in cardiomyocytes (Morimoto et al., 2008)or activates the autophagy by upregulating AMPK and JNK1 to alleviate the apoptosis of cardiomyocytes under ischemic stimulation (Yao et al., 2018). However, in our study, we did not confirm the therapeutic effect of curcumin directly in cardiomyocytes, which reflected by the fact that no significant change in cardiomyocyte apoptosis was found in curcumin treated group in mice heart 7 days after MI compared with MI only. In addition, previous studies have also reported that the administration of curcumin in ischemic diseases can salvage the functionality of endothelium (Pu et al., 2013), in our study, we also detected the quantity of CD31 and vWF in ischemic border area but demonstrated no significant difference in the presence or absence of curcumin 28 days after MI (data not shown). The findings illustrated above strongly hints that the regulation of cardiac fibrosis by curcumin may play pivotal role in improving cardiac function after MI. Although it has been well-validated that curcumin inhibits inflammation and anti-ROS as described previously, in a number of studies, curcumin was reported to be directly associated with collagen deposition and fibroblast proliferation. In ischemia-reperfusion (I/R) model, curcumin has been shown to function in regulating both ECM construction and anti-oxidative stress. The downregulated expression of TGF-β1 and *p*-SMAD2/3 and the upregulation of SMAD7 contributed to this therapeutic effect (Wang et al., 2012). To our surprise, we did not observe obvious inhibition of cardiac fibroblast *trans*-differentiation via direct treatment of curcumin with NRCF, and phosphorylated SMAD2/3 levels were found to be equal in the presence or absence of curcumin in NRCF, indicating that curcumin only inhibited cardiac fibrosis through macrophages in our study.

The TGF-β1/SMADs signaling pathway has been found to play an important role in inducing and exacerbating the pathological process of myocardial fibrosis after MI (Walton et al., 2017). Specifically, upon binding with the TGF-β1 receptor on the surface of myocardial fibroblasts, TGF-β1 stimulates phosphorylation of downstream SMADs protein (mainly SMAD2/3) and translocation into the nucleus in combination with SMAD4, induces myocardial fibroblast proliferation, phenotypic transformation, and collagen synthesis and ultimately promotes extracellular matrix formation and myocardial fibrosis. In our study, we demonstrated that cardiac fibroblasts received pro-fibrotic cytokines secreted from activated macrophages, thereby enhancing the expression level of IL-18, which in turn promotes phosphorylated SMAD2/3 and subsequent nuclear translocation of *p*-SMAD2/3, which could be inhibited by curcumin administration on macrophages.

This study had several limitations. First, we detected significantly decreased CD3^+^ cells in the acute phase post-MI; however, the effect of curcumin on CD3^+^ cells have not been clarified in this study, as T cells have relatively less connection with fibrosis procedure post injury; however, it is worth studying in the future. Second, the polarization of macrophages (monocytes) post-MI needs to be validated in future studies because both M1 type macrophages (which secrete IL-6, IL1β, and TNF-α) and M2 type macrophages, which serve as reparative forms post injury, can initiate cardiac fibrosis. Finally, because IL-18 serves mostly as a secreted cytokine to exert its biological function, it is to be identified whether IL-18 directly promotes SMAD2/3 nuclear translocation using a genetic knock-out/knock-in animal model.

In summary, our study revealed that the administration of curcumin significantly ameliorated inflammation in the acute phase, as reflected by the promotion of macrophage apoptosis, accompanied by decreased pro-inflammatory cytokine secretion, including IL-6, IL1b, and TNF-α. The alteration of macrophage status is subsequently linked with resident cardiac fibrosis, resulting in decreased expression of IL-18 in fibroblasts and hampered phosphorylation of SMAD2/3 in cardiac fibroblasts, reduced excessive collagen synthesis, and preserved long-term cardiac function post-MI. These findings suggest that curcumin has potential as a potent therapeutic target in treating adverse remodeling in ischemic heart disease.

## Data Availability

The datasets presented in this study can be found in online repositories. The names of the repository/repositories and accession number(s) can be found in the article/Supplementary Material.
