# Supplementary material for: Curcumin Ameliorates Cardiac Fibrosis by Regulating Macrophage-Fibroblast Crosstalk via IL18-P-SMAD2/3 Signaling Pathway Inhibition
Source: Front Pharmacol. 2022 Jan 18;12:784041. doi: 10.3389/fphar.2021.784041 (PMC8804383; doi:10.3389/fphar.2021.784041)
Supplement: Supplementary file 1 [file DataSheet1.DOCX]

**Supplemental Material**

Curcumin ameliorates cardiac fibrosis by regulating macrophage-fibroblast crosstalk via IL18-p-SMAD2/3 signaling pathway inhibition


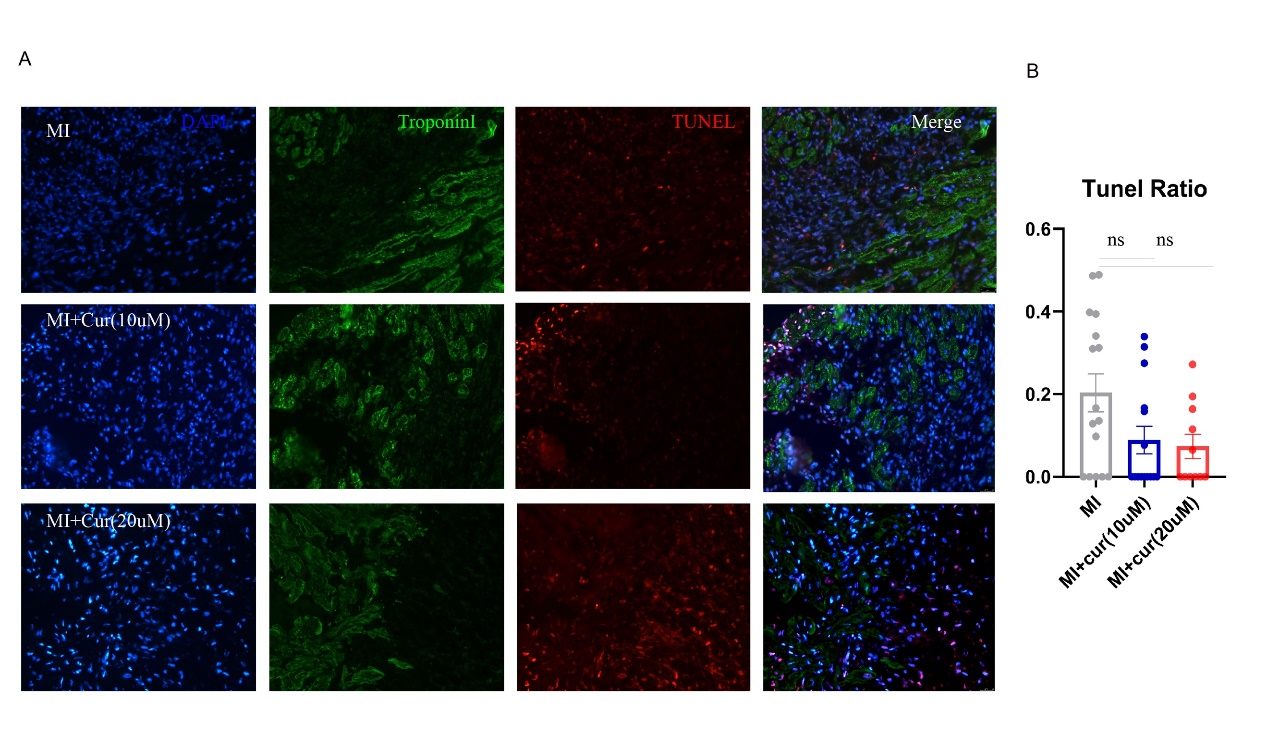


**Supplemental Figure 1. Cardiomyocyte apoptosis within peri-infarct zone in rat post-MI**

**A-B).** Apoptosis counts reflected by TUNEL staining (highlighted red) in peri-infarct heart samples harvested 28 days post-MI from SD rats in the MI, MI+ Cur 50 mg/kg, MI+ Cur 100 mg/kg). Troponin was identified by green coloring, and apoptosis in cardiomyocytes was determined by overlapped-TUNEL with troponin. The statistical data are summarized in **B).** n=7-10 in each group**.** Results are mean with SEM; NS=no significance between groups.


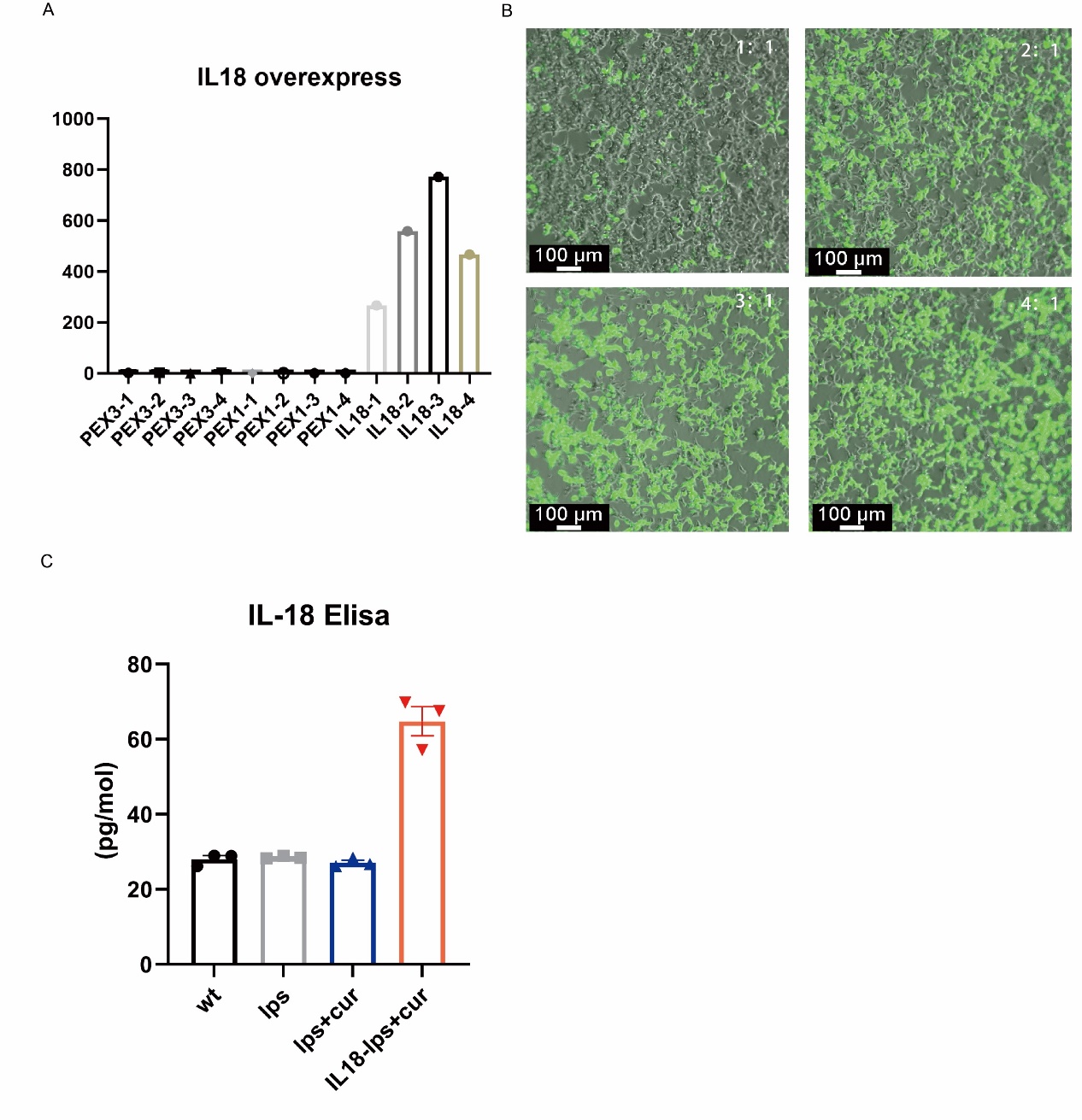


**Supplemental Figure 2. IL-18 transfection efficacy in neonatal rat cardiac fibroblast**

**A).** RT-PCR results reflected overexpression efficacy in gene-level alterations with different IL-18 overexpression plasmids. **B).** GFP staining indicated the percentage of NRCF that was transfected. **C)**. ELISA detected significantly upregulated IL-18 protein expression in the NRCF cell supernatant.
